# The emergence of lipid droplets in yeast: current status and experimental approaches

**DOI:** 10.1007/s00294-013-0407-9

**Published:** 2013-09-21

**Authors:** Maja Radulovic, Oskar Knittelfelder, Alvaro Cristobal-Sarramian, Dagmar Kolb, Heimo Wolinski, Sepp D. Kohlwein

**Affiliations:** 1 Institute of Molecular Biosciences, University of Graz, Humboldtstrasse 50/II, 8010 Graz, Austria; 2 Institute of Cell Biology, Histology and Embryology and Center for Medical Research, Medical University of Graz, 8010 Graz, Austria

**Keywords:** Neutral lipid homeostasis, Triacylglycerol, Fluorescence microscopy, CARS microscopy, Electron microscopy, Electron tomography, Lipidomics

## Abstract

The ‘discovery’ of lipid droplets as a metabolically highly active subcellular organelle has sparked great scientific interest in its research in recent years. The previous view of a rather inert storage pool of neutral lipids—triacylglycerol and sterols or steryl esters—has markedly changed. Driven by the endemic dimensions of lipid-associated disorders on the one hand, and the promising biotechnological application to generate oils (‘biodiesel’) from single-celled organisms on the other, multiple model organisms are exploited in basic and applied research to develop a better understanding of biogenesis and metabolism of this organelle. This article summarizes the current status of LD research in yeast and experimental approaches to obtain insight into the regulatory and structural components driving lipid droplet formation and their physiological and pathophysiological roles in lipid homeostasis.

## Lipid droplets: an introduction

Virtually all eukaryotic cells have the capacity to form and sequester neutral lipids within lipid droplets (LDs) in the cytosol, especially when exposed to high levels of nutritional fatty acids. In addition to their function to store the so-called neutral lipids—triacylglycerols, sterols and/or steryl esters—, LDs contribute to diverse cellular functions (Farese and Walther 2011; Kohlwein et al. 2013; Walther and Farese 2012; Zechner et al. 2012), which include signaling, temporal protein storage and protein degradation (Fujimoto et al. 2008; Murphy 2001; Murphy et al. 2009). In addition to their role in lipid storage and signaling, multiple pathophysiological roles of LDs continue to emerge, for instance as regulators of viral replication (Miyanari et al. 2007), atherosclerosis (Faber et al. 2001), and even cancer (Accioly et al. 2008). In yeasts, LDs may also function as a depot for non-natural and potentially harmful sterols that are taken up from the environment (Taylor and Parks 1981; Valachovič et al. 2001). The biotechnological exploitation of microorganisms as potential sources for biodiesel (Liang and Jiang 2013) or high value polyunsaturated fatty acids for nutritional purposes is a further driving force in developing a better understanding of the processes contributing to lipid storage and LD formation.

**Fig. 1:**
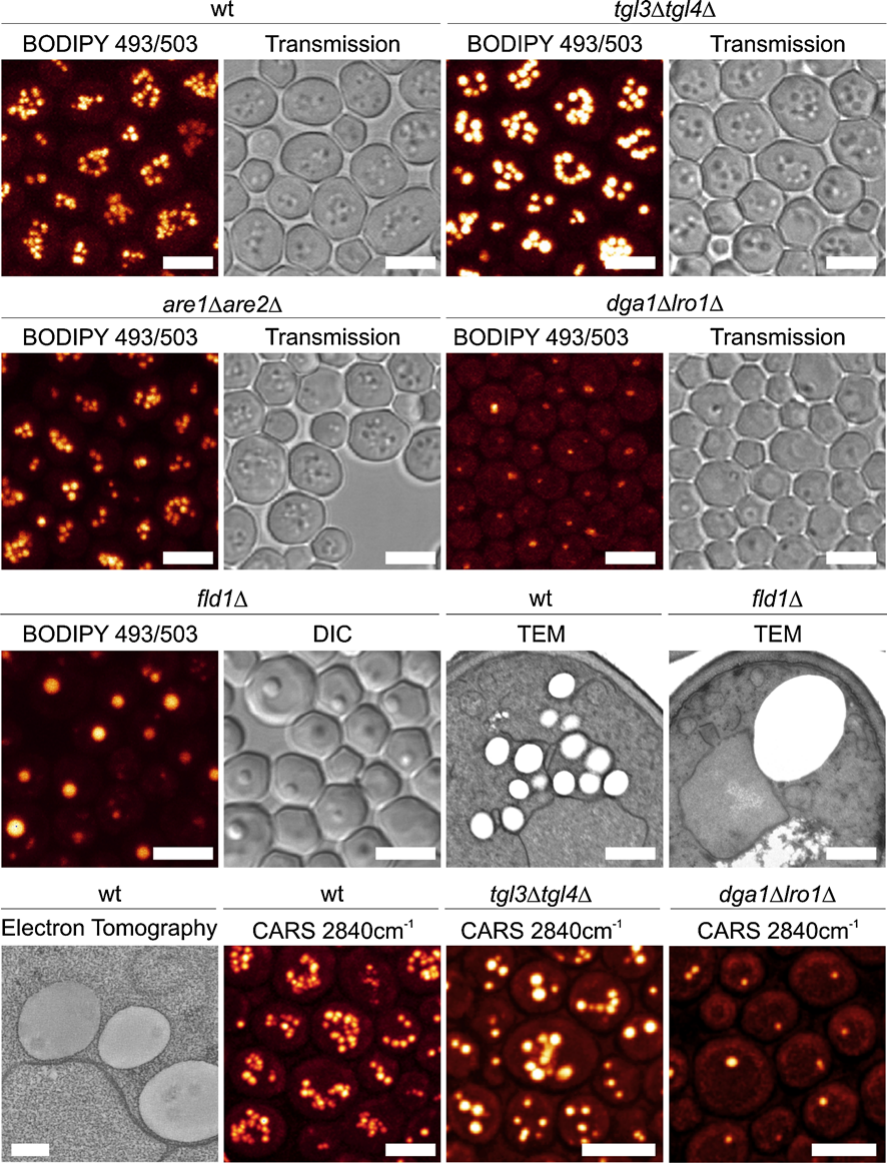
Fluorescence, CARS and electron microscopy/tomography of lipid droplets in yeast wild type and mutant cells [reproduced from (Kohlwein et al. 2013), with permission; ©Genetics Society of America]. *tgl3 tgl4* mutants lack the major TAG lipases, which result in LD accumulation; *dga1 lro1* mutants lack the major acyltransferases involved in TAG synthesis, the remaining LD(s) contain steryl esters only; *are1 are2* mutants are deficient in steryl ester production, but there is very little impact on number and size of LD in these strains, which are composed of TAG only. *fld1* mutants lack the yeast ortholog of seipin, a protein of unknown function that is implicated in lipodystrophy in humans. *DIC* differential interference contrast, *CARS* coherent anti-stokes Raman scattering microscopy. See text for details. *Scale bar* = 5 μm in fluorescence images and 0.2 μm in electron microscopy images

The yeast *Saccharomyces cerevisiae* is a well-established experimental model organism and has proven also very valuable in understanding lipid synthesis and its regulation. Great efforts have also been directed in recent years toward exploiting yeast as a system to better understand the mechanisms underlying LD formation [see (Kohlwein et al. 2013) for review]. The detailed knowledge about biosynthetic pathways in yeast (Henry et al. 2012) combined with an ever-expanding tool box for biochemical and genetic manipulation has created a great momentum in yeast LD research (Kohlwein 2010; Kohlwein et al. 2013). These studies are also facilitated by the ease of detecting and analyzing LDs in living and fixed cells by microscopic techniques (Fig. 1; for details see below).

Yeast provides multiple experimental advantages for studying LD biogenesis in greater detail: (1) the core structural organization of yeast cells resembles a typical eukaryotic cell with all relevant organelles in place; (2) highly conserved biochemical pathways in lipid metabolism and its regulation; (3) the ease of biochemical manipulation, e.g. by supplementing exogenous fatty acids driving TAG and LD formation; (4) an ever increasing repertoire of methods to identify genes and proteins involved in these processes; (5) well-established LD isolation protocols for structural studies in vitro; (6) a rather simple lipid composition; (7) large-scale approaches to identify mutants with defective LD morphology, including imaging-based screens.

## Lipid metabolic pathways driving lipid droplet formation

**Fig. 2:**
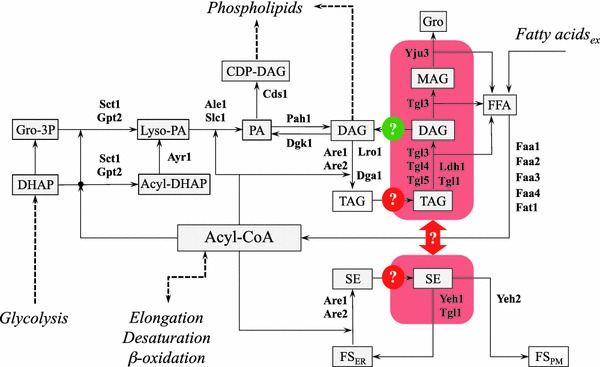
Pathways of neutral lipid metabolism in yeast (Henry et al. 2012; Kohlwein et al. 2013). *Red areas* mark lipid droplets. Whether steryl ester and triacylglycerol form distinct or mixed LDs is currently unknown. The mechanisms by which TAGs (that are mostly) and SE (that are exclusively) generated in the ER enter the LD are unknown. It is also unclear whether and to what extent DAG derived form lipolysis is directly utilized for re-acylation or for phospholipid synthesis; the stereochemistry of the lipolysis reaction in yeast has not yet been worked out. *Gro* glycerol, *DHAP* dihydroxyacetone phosphate, *Gro-3P* glycerol-3-phosphate, *Lyso-PA* sn1-acyl-gycerol-3-phosphate (lyso phosphatidic acid), *PA* phosphatidic acid, *DAG* diacylgycerol, *MAG* monoacylglycerol, *SE* steryl esters, *FFA* free fatty acids, *FS* free sterol, *ER* endoplasmic reticulum, *PM* plasma membrane. (See text for details)

The enzymology of (neutral) lipid synthesis has been very well worked out in the yeast *S. cerevisiae* [see (Henry et al. 2012; Kohlwein et al. 2013) for recent reviews]; however, the mechanisms involved in LD formation are currently unknown and several models have been put forward to explain the presence of a monolayer of phospholipids, which delineates the LD surface. All these models have in common that LDs are derived from the endoplasmic reticulum (ER), which harbors also most of the enzymes involved in the formation of the neutral lipid core (Kohlwein et al. 2013). Figure 2 summarizes the biochemical pathways associated with the formation of neutral lipids, triacylglycerol (TAG) and steryl esters (SE), which form the core of the LD. Thus, processes affecting neutral lipid homeostasis can be potentially identified by morphological alterations of lipid droplet structure(s); conversely, mechanisms driving LD formation likely also regulate the synthesis of LD lipids. Noteworthy, the initial pathway of TAG synthesis up to phosphatidic acid is shared with the biosynthesis of the ‘de novo’ branch of phospholipid synthesis, which leads to all major phospholipid classes under normal growth conditions (Henry et al. 2012). In addition, diacylglycerol, which is generated (among other pathways) by dephosphorylation of phosphatidic acid by the conserved enzyme phosphatidate phosphatase (Pah1 in yeast, Lipin in mammals), serves as a phospholipid precursor, by utilizing ethanolamine or choline precursors derived from exogenous sources or from internal phospholipid turnover to form phosphatidylethanolamine or phosphatidylcholine, respectively. Thus, it is not surprising that processes affecting TAG homeostasis also affect phospholipid metabolism and, as a consequence, membrane function. Conversely, defective phospholipid synthesis and turnover also trigger TAG accumulation (Gaspar et al. 2008; Malanovic et al. 2008). This close metabolic interrelationship between TAG synthesis and membrane lipid composition and function may be the underlying cause of lipotoxic cell damage, which further underscores the importance of obtaining a clearer picture of the mechanisms of TAG storage into LD.

## Localization and topology of enzymes involved in neutral lipid homeostasis

One important aspect in understanding the nature of the ER–LD connection is the development of a better view on the localization and topology of yeast acyltransferases driving TAG and SE synthesis. Most of the enzymes involved are associated with the ER or cytosol (Kohlwein et al. 2013). Pah1, the yeast phosphatidic acid phosphatase is catalyzing the committed step in TAG synthesis and changes its subcellular localization depending on its phosphorylation state, from cytosolic (phosphorylated) to ER-associated (Adeyo et al. 2011; Choi et al. 2011). This reaction is considered the key regulator for the production of TAG (Carman and Henry 2007) and, hence, LD formation. DAG generated by the Pah1 reaction is the substrate for Lro1, the yeast ortholog of mammalian lecithin:cholesterol acyltransferase (LCAT), which localizes to the ER with its active site exposed to the lumen (Jacquier et al. 2011). The second major acyltransferase involved in TAG synthesis, Dga1 (the ortholog of mammalian DGAT2), localizes mostly to the ER and also associates with LDs (Dahlqvist et al. 2000). Yeast mutants lacking both Lro1 and Dga1 still contain LDs which are composed of steryl esters only, and only the additional elimination of the Are1 and Are2 acyltransferases that catalyze sterol acylation completely abolishes LD formation [see (Kohlwein 2010; Kohlwein et al. 2013) for reviews]. These *dga1 lro1 are1 are2* quadruple mutants are viable, indicating that LD formation is not an essential process under normal growth conditions (Sandager et al. 2002). However, the ability to synthesize TAG and LDs becomes essential in the presence of excess (unsaturated) fatty acids, which trigger mitochondria-dependent necrotic cell death (Garbarino et al. 2009; Garbarino and Sturley 2009; Kohlwein and Petschnigg 2007; Petschnigg et al. 2009; Rockenfeller et al. 2010). Interestingly, a soluble, cytosolic diacylglycerol acyltransferase involved in TAG biosynthesis was recently identified in *Rhodotorula* *glutinis*, which appears to complement the LD defect in yeast mutants lacking these enzymes (Rani et al. 2013). How this cytosolic TAG production may contribute to LD formation remains to be determined.

Notably, most conserved motifs containing the presumed active sites of 1-acyl-sn-glycerol-3-phosphate acyltransferases Slc1 and Ale1 and the related membrane-bound *O*-acyltransferases (MBOATs) of *S.* *cerevisiae* are oriented toward the ER lumen. This strongly suggests that the biosynthesis of phosphatidic acid in yeast may occur on the luminal side of the ER (Pagac et al. 2011). Topological analysis of yeast acyl-CoA: diacyglycerol acyltransferase Dga1 provides evidence that both the N and C termini are oriented toward the cytosol and have different catalytic roles (Liu et al. 2011).

Degradation of TAG occurs on the LD surface where the three major TAG lipases Tgl3, Tgl4, Tgl5 are localized (Athenstaedt and Daum 2003; Athenstaedt and Daum 2005; Kurat et al. 2006). Recent data show that in the absence of LDs, the major TAG lipase Tgl3 re-localizes to the ER where it may be stored in a ‘parking position’ without enzymatic function (Schmidt et al. 2013). The second major components of LDs, steryl esters (SE) are degraded by the SE hydrolases Tgl1 and Yeh1 on LDs (Jandrositz et al. 2005; Koffel et al. 2005) and by Yeh2 at the plasma membrane (Koffel et al. 2005; Müllner et al. 2005). Notably, biosynthetic enzymes typically localize to both the ER and LDs, consistent with the hypothesis that LDs emerge from the ER, whereas catabolic enzymes are largely restricted to LDs and appear only in the ER (or cytosol) in the absence of LD formation. The question as to the specific protein targeting signals driving their LD association is still elusive. Since neutral lipid synthesis is tightly coupled to LD formation, more detailed information about the spatial organization and topology of the enzymes involved is required. For instance, the question as to how transmembrane segments of ER-resident proteins are accommodated in the phospholipid monolayer of the LD surface remains to be solved and is subject of ongoing studies.

## Experimental approaches to lipid droplet biology

### Isolation of lipid droplets

Structural and functional analyses of LDs require a versatile protocol for their isolation to the highest possible purity. Since LD metabolism is subject to substantial dynamics in growing cells, it is critical to choose the proper time point for their isolation, to obtain maximum yield. On the other hand, this restricts the analysis of LDs in vitro to specific time points in the yeast growth cycle, and information about dynamic alterations of the LD lipidome and proteome are lost. Since LDs are intimately associated with other organelles as part of their physiological role in controlling lipid fluxes, cross contamination during isolation is almost inevitable. A further major limitation is the massive cell wall, which requires either mechanical disruption or enzymatic degradation and, again, determines to a large extent yield and purity of the LD fraction (Connerth et al. 2010b; Leber et al. 1994; Zinser and Daum 1995; Zweytick et al. 2000a). With these current protocols, the purity of LD fractions is high but the procedure is quite time consuming. LDs are typically isolated by flotation, involving several gradient centrifugation steps. A major contaminant in LD isolation protocols are vacuoles, which tend to stick to LDs and co-flotate on the gradient used for isolation. This can be resolved by additional buffer changes and repeated centrifugation to break the interaction with the vacuolar membrane, which can be collected at the bottom of the tube. Starting from a culture volume of 5 L, current high-purification protocols typically yield about 600 μg of LD protein, which can be subjected to lipidomics and proteomics analyses (see below). Recently, Ding et al. (2013) published a more rapid rather small-scale LD isolation protocol, which yields about 25 μg of LD protein from 400 mL culture volume.

### Structural analysis of lipid droplets in vitro

LDs of all eukaryotic species have a similar and rather simple structure: they consist of a highly hydrophobic, nonpolar core of neutral lipids surrounded by a monolayer of polar lipids that delineates the LD surface. Numerous proteins, mainly involved in lipid metabolism and its regulation, are associated with or embedded into the phospholipid monolayer (see below). Structural characterization of isolated LDs from various yeast mutant strains has been performed by differential scanning calorimetry and X-ray analysis. These studies have unveiled that LD contains rigidly packed shells of SE on the surface of the neutral lipid core that becomes fluid-like at certain transition temperatures (Czabany et al. 2008). Reversible changes of the structure upon heating and cooling indicated that not all SE in LD were in an ordered state but perturbed by intercalated TAG. SE only and TAG only LDs differ somewhat in their sizes, and although the biophysical characterization performed so far indicates a structured organization of the LDs containing both types of neutral lipids, information on their concerted assembly and regulated degradation is lacking. Coherent anti-stokes Raman scattering (CARS) or stimulated Raman scattering (SRS) microscopy (see below) may be the suitable spectroscopic imaging techniques to differentiate between TAG and SE content in individual LDs in vivo.

### Lipid droplets: proteome and lipidome

The inventory of yeast lipid droplets is rather simple. Using highly purified LDs and mass spectrometry, Athenstaedt et al. (1999) have pioneered the analysis of the yeast LD proteome, which has been refined ever since (Grillitsch et al. 2011; Natter et al. 2005); [see (Kohlwein et al. 2013) for review]. Notably, most of the identified proteins appear to have a function in lipid metabolism; however, multiple LD-associated proteins have not been functionally characterized yet and often co-localize with other subcellular organelles. Enzymes of known function residing on LDs are involved in fatty acid activation, ergosterol biosynthesis, and lipid de novo synthesis and hydrolysis (Athenstaedt et al. 1999; Grillitsch et al. 2011; Kohlwein et al. 2013; Zweytick et al. 2000a). The protein distribution on LDs is also subject to major changes depending on the carbon source (Grillitsch et al. 2011); in addition, size and phospholipid composition may influence the LD proteome (Fei et al. 2011b). It needs to be stressed that multiple LD proteins show a dual localization and are also present in other organelles, mostly in the ER. This observation not only underscores the close physical and functional relationship between these two organelles, but also raises the question as to the physiological relevance of the LD association of these proteins (see “Isolation of lipid droplets”).

Table 1 summarizes data for the lipid components of LDs isolated from cells grown on rich media with glucose or oleic acid as the sole carbon source. For this analysis, lipids were extracted from highly purified LDs following standard protocols (Schneiter and Daum 2006a, b) and subjected to quantitative thin layer chromatography or electro-spray ionization mass spectrometry (ESI–MS) methods (Ejsing et al. 2009; Schneiter and Daum 2006a). The phospholipid content of yeast LDs is rather low, consistent with the monolayer structure delineating the LD surface. Phosphatidylcholine (PC) is the predominant phospholipid class, which reflects its overall abundance in cellular phospholipids; phosphatidylinositol (PI) appears somewhat enriched in the LD fraction over the ER or other subcellular membranes (Connerth et al. 2010b; Grillitsch et al. 2011; Schneiter et al. 1999). Phosphatidylserine, cardiolipin and dimethyl-phosphatidylethanolamine (a precursor of phosphatidylcholine) can also be detected in isolated LDs, but their content may vary, depending on growth on either glucose or oleic acid as the sole carbon sources (Grillitsch et al. 2011). Cardiolipin is a mitochondrion-specific phospholipid and its appearance in the LD fraction may indicate some level of cross contamination. The presence of the fusogenic phospholipids phosphatidylethanolamine (PE) and phosphatidic acid (PA) is thought to contribute to the formation of so-called supersized lipid droplets (SLDs) (Fei et al. 2011a). In general, the phospholipid monolayer of LDs is enriched in di-unsaturated molecular species compared to other subcellular membranes (Schneiter et al. 1999).

**Table 1:** Lipid composition of lipid droplets in comparison with total cell extracts in *S.* *cerevisiae* grown on different carbon sources

	Glucose		Oleate	
	Lipid droplets (mg/mg protein)	Homogenate (mg/mg protein)	Lipid droplets (mg/mg protein)	Homogenate (mg/mg protein)
TAG	n.a.	32.0 ± 4.0	n.a.	97.3 ± 8.9
SE	n.a.	36.7 ± 4.1	n.a.	1.0 ± 0.3
Phospholipids	0.423 ± 0.048	0.047 ± 0.003	0.889 ± 0.054	0.071 ± 0.004
% of total phospholipids				
PA	1.8 ± 1.3	2.8 ± 0.4	1.3 ± 2.7	0.7 ± 0.7
PI	21.5 ± 3.4	14.5 ± 5.9	21.5 ± 3.4	16.9 ± 3.8
PS	2.1 ± 2.6	3.8 ± 0.4	0.8 ± 0.9	3.3 ± 0.9
PC	57.5 ± 1.7	51.5 ± 5.5	56.4 ± 2.7	53.0 ± 1.4
PE	16.6 ± 1.9	23.6 ± 1.4	16.9 ± 2.8	20.1 ± 3.7
CL	0 ± 0	2.3 ± 0.3	1.0 ± 1.2	3.7 ± 0.8
LP	0.3 ± 0.6	0 ± 0	0.7 ± 0.5	0.3 ± 0.6
DMPE	0 ± 0	1.9 ± 1.2	1.3 ± 1.9	1.5 ± 1.4

Data from Grillitsch et al. (2011)

*n.a* Data not available

In cells growing on glucose, TAG and SE are present in about equal amounts, but this ratio is drastically shifted when cells are grown on oleic acid-containing media (in the absence of glucose) (Connerth et al. 2010a; Grillitsch et al. 2011; Leber et al. 1994; Zinser et al. 1993). Cellular TAG content triples, whereas SE content is markedly reduced, presumably due to the inhibition of Are activity by free fatty acids (Connerth et al. 2010a; Grillitsch et al. 2011). The composition of TAG molecular species resembles the cellular distribution of saturated and unsaturated long chain fatty acids (C16, C18) (Connerth et al. 2010a; Grillitsch et al. 2011); however, the distribution of TAG molecular species changes drastically, depending on the carbon source. Upon growth on oleic acid, this fatty acid is also most prominently represented in the TAG pattern (i.e. increase in TAG 54:3 molecular species, i.e. glycerol esterified with three oleoyl chains) (Grillitsch et al. 2011). Steryl esters contain mostly unsaturated fatty acids (palmitoleic and oleic acid) esterified to ergosterol (Czabany et al. 2008). Sterol intermediates such as zymosterol, epiestriol and fecosterol can also be found in the SE fraction of LDs (Czabany et al. 2008; Zweytick et al. 2000b).

### Ultrastructural analysis of lipid droplets: electron microscopy and electron tomography

Currently, the most effective way of obtaining morphological information about LDs and their interaction with other subcellular organelles and structures is by electron microscopy, preferably by combining high pressure freezing (HPF) with electron tomography. The combination of these techniques enables the visualization of a process at a specific point in time in three dimensions and as close as possible to the native state. With HPF and freeze substitution, it is possible to visualize the interaction of LD with ER membranes without the artifacts that are potentially introduced by chemical fixation, e.g. extraction and/or condensation of proteins and structural distortions. With HPF, fresh yeast cells, which are still surrounded by growth medium, are flash frozen under 2,000 bar in liquid nitrogen within milliseconds, thus avoiding the formation of ice crystals which may otherwise destroy subcellular structures (‘vitreous ice’). Freeze substitution and contrasting at a temperature below −70 °C maintain the frozen state until samples are embedded in resin and sectioned (Studer et al. 2008). For conventional electron microscopy, typically 70 nm sections are investigated, and thicker sections of up to 300 nm are used for electron tomography, to obtain extended three-dimensional information. Since the initial process of LD formation is currently unknown, electron tomography of yeast mutant strains with de-regulated LD formation holds great promise for obtaining a better understanding of the mechanisms involved in their biogenesis.

### Lipid droplet morphology and dynamics in vivo

Lipid droplets can be readily visualized in living cells using various fluorescence or spectroscopy-based techniques, which significantly expand the experimental possibilities to study LD formation, morphology, interaction with other organelles and their turnover. Microscopic techniques not only allow the virtually non-invasive observation of dynamic changes of the LD morphology itself, but also potentially the flux of proteins and lipids and their association with LDs. These techniques not only aim at depicting LDs, but also are increasingly powerful to extract quantitative information about organelle morphology and dynamics. In addition, the combination of mutant collections, compound libraries and imaging-based screens opens unprecedented possibilities for the discovery of LD-associated processes.

#### Microscopic analysis of lipid droplets

A number of fluorescence dyes are available for labeling yeast LDs (Table 2). LDs can be either directly labeled using reference dyes or are stained by incorporation of fluorescently labeled fatty acid analogs. Nile red (Greenspan and Fowler 1985; Greenspan et al. 1985) has been used to identify LDs in yeast cells using fluorescence microscopy (Kohlwein 2000) or to estimate neutral lipid content using fluorescence microplate readers (Sitepu et al. 2012). Nile red is suitable for the simultaneous detection of green-fluorescent proteins (GFP) and LDs in multi-color approaches (Kurat et al. 2006; Wolinski and Kohlwein 2008; Wolinski et al. 2009a; Wolinski et al. 2011). However, the dye shows significant solvatochromism (Greenspan and Fowler 1985) and changes its fluorescence characteristics depending on its environment. Thus, optimized microscope setup is required to avoid spectral overlap between Nile red and GFP fluorescence emission. LD540 (Spandl et al. 2009) provides an alternative to Nile red for LD detection; it shows improved spectral discrimination between green and red fluorophores and thus facilitates imaging of multi-labeled specimens in different cell systems, including yeast (Wolinski et al. 2011). In addition, LD540 shows better photostability and less photo bleaching than Nile red (Spandl et al. 2009). However, at higher concentrations and upon intense light illumination, the dye may induce LD fusion (Wolinski unpublished observation). BODIPY™ 493/503 (Invitrogen, Inc., USA) is a green emitting fluorophore for high-contrast labeling of yeast LD. It shows very high quantum yield and photostability. Furthermore, the fluorescence dye diffuses more readily into living yeast cells compared to Nile red and LD540. Thus, BODIPY™ 493/503 staining of neutral lipids is the method of choice when GFP co-labeling is not required (Wolinski et al. 2012b). Extracellular quenching of BODIPY™ 493/503 fluorescence facilitates the use of fluorescence microplate readers for the estimation of LD content in cell populations (Bozaquel-Morais et al. 2010). Moreover, BODIPY™ 493/503 is compatible with red-emitting fusion proteins such as mCherry or dsRed (Matz et al. 1999; Shaner et al. 2004). BODIPY™ 493/503 shows to some extent solvatochromism and also labels the more polar membrane phospholipids. The intensity of phospholipid labeling, however, largely depends on the amount of neural lipids in the cells with which the dye can equilibrate. This phenomenon limits the detection of small developing LDs due to the increased ‘background’ fluorescence of membranes. On the other hand, the increased affinity of Nile red and BODIPY™ 493/503 to polar environments in the absence of LDs has been utilized to identify accumulation of subcellular membranes in specific mutant strains (Adeyo et al. 2011; Petschnigg et al. 2009). It should also be noted that fluorescence dyes may be actively and efficiently pumped out of yeast cells by pleiotropic drug resistance pumps (Ivnitski-Steele et al. 2009; Wolinski et al. 2009a). Preparation techniques have been developed to overcome this problem, particularly for long-term cell observations and four-dimensional live cell imaging (see below). In addition, dye uptake also depends on cellular age and growth stage, which may result in heterogeneous labeling of cell populations. These dyes are compatible with cross-linking fixatives such as formaldehyde, and fixation typically results in more homogenous labeling of LDs in yeast cell populations (Wolinski and Kohlwein 2008). Specific and high-contrast labeling of yeast LD is a prerequisite for image-based quantitative analysis and for estimation of the LD content in yeast cells (Wolinski et al. 2012a). Other promising neutral lipid-specific fluorescence dyes such as monodansylpentane [blue emission; (Yang et al. 2012)] or LipidTox (red and deep red emission; Invitrogen, Inc., USA) have not been tested in yeast cells so far. In addition to the reference dyes mentioned above, BODIPY™-labeled fatty acid analogs are useful for labeling yeast LD. Green emitting acyl chain or head group-labeled fatty acids (BODIPY-C12, C1-BODIPY-C12; Invitrogen, Inc., USA) have been used not only for monitoring the uptake and incorporation of fatty acids into yeast cells and LD (DiRusso et al. 2000; Faergeman et al. 2001; Jacquier and Schneiter 2010), but also for high-throughput screens for fatty acid uptake inhibitors (Li et al. 2008). The red fluorescent version of BODIPY-C12, BODIPY™ 558/568, is compatible with GFP detection (Wolinski et al. 2012a).

**Table 2:** Commonly used fluorescent dyes for yeast lipid droplet microscopy

Dye	*λ*_ex_ (nm)	*λ*_em_ (nm)	Remarks	Reference/source
BODIPY™ 493/503	493	500–530	High quantum yield	Invitrogen Inc., USA
Nile red	488 or 543	550–560: LD	Shows solvatochromism and broad excitation and emission spectra; strong fluorescence bleaching	Greenspan et al. (1985), Wolinski et al. (2009a), Petschnigg et al. (2009)
	488 or 543	600–650: LD and phospholipids		
LD540	543	550–600	Compatible with GFP detection; high quantum yield	Spandl et al. (2009) Wolinski et al. (2012b)
	561	565–600		
BODIPY™ 558/568-C12	543	550–600	Compatible with GFP detection; uptake depends on cell physiology	Invitrogen Inc., USA
	561	570–600		
Oil red O	543 or 561	550–570 or 570–650	Cell impermeable and requires fixation; alternative to fluorescence: analysis of the red color (absorption) in bright field images	Adeyo et al. (2011)

An alternative to fluorescence dyes to analyze LD is the spectroscopic imaging technique, CARS, which has recently made it out of the physics laboratories to the commercial market and may be implemented in standard confocal microscope systems. CARS is a label-free imaging technique based on molecular vibrations that are excited with IR light (Evans and Xie 2008; Le et al. 2010; Muller and Zumbusch 2007). C–H vibrations, which are particularly abundant in the acyl chains of lipids, are excited in a multi-photon process and stimulated to emit blue-shifted light, which can be detected. This allows for a label-free observation particularly of LDs in living cells over extended periods of time, due to the benign nature of the IR light source that is used in that process. This technique has been successfully applied to detect LDs in living yeast cells (Chumnanpuen et al. 2012; Kohlwein 2010; Kohlwein et al. 2013) (see also Fig. 1). In addition, CARS microscopy allows monitoring and ratio-imaging of the uptake and metabolism of deuterium labeled fatty acids in living yeast cells due to the specific IR absorption band of the C-D vibration (Wolinski et al., unpublished). Pitfalls and limitations of this new technique for yeast live cell imaging, e.g. long-term effect of the intense laser power required to generate vibrational contrast on cell physiology and organelle integrity, remain to be investigated.

#### Analyzing lipid droplet dynamics: FRAP and FLIP and photoactivatable fluorescent proteins

The turnover and dynamics of LD-associated proteins can be addressed qualitatively and quantitatively using GFP fusion proteins and bleaching techniques (Lippincott-Schwartz and Patterson 2003; Snapp et al. 2003). Fluorescence recovery after photobleaching (FRAP) and fluorescence loss in photobleaching (FLIP) of GFP-tagged proteins indicate a continuum between the ER and the LD surface, which allows proteins (and perhaps phospholipids) to diffuse and translocate between these organelles (Jacquier et al. 2011). Photoactivatable or photoconvertible fluorescent protein fusions may also be used to study protein dynamics in intact yeast cells; indeed, light-induced activation of the photoactivatable GFP (PA-GFP) fused to Erg6 involved in ergosterol biosynthesis has already been demonstrated in yeast (Vorvis et al. 2008).

#### Imaging-based approaches to lipid droplet biology

Yeast provides an excellent experimental system for imaging-based genome-wide screens. Determination of the subcellular localization of more than 600 GFP-tagged yeast proteins involved in lipid metabolism has provided new insights into the spatial organization of these processes in living cells (Natter et al. 2005). This analysis has uncovered multiple novel LD-associated proteins, which have escaped proteomics analysis of isolated LDs. This underscores the power of in vivo imaging approaches, which enable identification of dynamic and transient LD associations. High-resolution image data derived from a large-scale confocal microscopy study of the yeast GFP collection (Huh et al. 2003), currently representing about 3800 GFP-expressing strains (YPL+, http://yplp.uni-graz.at/), has been integrated into the saccharomyces genome database (SGD, Stanford University). Targeted imaging-based screens of the yeast deletion mutant collection for aberrant morphology LDs using fluorescent dyes yielded a large number of factors regulating LD content, formation, and inheritance (Fei et al. 2008; Fei and Yang 2012; Szymanski et al. 2007). These approaches led to the identification of the yeast ortholog of the human BSCL2 lipodystrophy protein, seipin (Fld1)(Fei et al. 2008; Szymanski et al. 2007), phosphatidic acid hydrolase (Pah1) responsible for the conversion of phosphatidic acid into diacylglycerol (Adeyo et al. 2011) and protein phosphatases regulating neutral lipid content (Bozaquel-Morais et al. 2010).

Alternative approaches using GFP-tagged LD proteins as reporters instead of fluorescent dyes have also been used. Organelle-specific GFP fusions are integrated into the mutant collection, using the synthetic genetic array technology (Li et al. 2011; Tong and Boone 2006; Tong et al. 2001; Vizeacoumar et al. 2009, 2010; Wolinski et al. 2012a). This strategy not only provides insights into mechanisms regulating LD biogenesis and morphology, but may also unveil currently unknown mechanisms for the targeting of distinct proteins to the LD.

Despite the small size of yeast cells (about one order of magnitude above the theoretical resolution limit of optical microscopy), ease of preparation, excellent statistics by looking at 100s of cells at the same time, and multiple levels of biochemical and genetic manipulation, application of modern imaging techniques holds great promise for advancing our understanding of cellular and molecular processes. Methodological advances in cell preparation and image acquisition as wells as sophisticated software and database systems enable reliable and user-friendly analysis of image-based screening data and facilitate their quantitative assessment and interpretation (Carpenter et al. 2006; Wolinski et al. 2009b; Bredies and Wolinski 2011; Vizeacoumar et al. 2010).

## Open questions, outlook

Despite the significant progress in understanding the biochemistry of neutral lipid synthesis and its regulation, major questions as to their packaging and assembly into mature LDs remain to be solved. In particular, the early stages of LD development, presumably at specific sites of the ER, have not been resolved yet, which should clarify the mechanisms by which LDs acquire their phospholipid monolayer.

In contrast to mammalian cells, yeast LDs do not contain perilipins or related proteins, which cover LDs in mammalian cells and regulate access of metabolic enzymes. What are the specific structural requirements for proteins to associate with LDs? How is this access regulated? Furthermore, the topology of LD-associated proteins, especially those containing (multiple) membrane spanning domains which need to be somehow accommodated in the LD surface, is yet to be clarified. More than any other membrane delineated organelle, biophysical properties of the lipid droplet core need to be considered when studying LD formation. Is the LD a ‘droplet’ i.e. liquid under physiological conditions, or rather a ‘particle’, which implies a more solid structure? Does LD fusion take place? How might this process be regulated to maintain a fairly uniform LD size distribution in the cells?

Current efforts are directed toward improved isolation protocols for refined in vitro studies—proteomics, lipidomics—as well as the implementation of sophisticated in vivo techniques such as CARS or photo-switchable fluorescent dyes, which will provide better insight into dynamic processes of LD biogenesis. LDs are emerging as fascinating ‘novel’ organelles that continue to be an experimental challenge, at the multidisciplinary interface between biochemistry, cell biology and biophysics.

## References

[CR1] Accioly MT, Pacheco P, Maya-Monteiro CM, Carrossini N, Robbs BK, Oliveira SS, Kaufmann C, Morgado-Diaz Ja, Bozza PT, Viola JPB (2008). Lipid bodies are reservoirs of cyclooxygenase-2 and sites of prostaglandin-E2 synthesis in colon cancer cells. Cancer Res.

[CR2] Adeyo O, Horn PJ, Lee S, Binns DD, Chandrahas A, Chapman KD, Goodman JM (2011). The yeast lipin orthologue Pah1p is important for biogenesis of lipid droplets. J Cell Biol.

[CR3] Athenstaedt K, Daum G (2003). YMR313c/TGL3 encodes a novel triacylglycerol lipase located in lipid particles of Saccharomyces cerevisiae. J Biol Chem.

[CR4] Athenstaedt K, Daum G (2005). Tgl4p and Tgl5p, two triacylglycerol lipases of the yeast Saccharomyces cerevisiae are localized to lipid particles. J Biol Chem.

[CR5] Athenstaedt K, Zweytick D, Jandrositz a, Kohlwein SD, Daum G (1999). Identification and characterization of major lipid particle proteins of the yeast Saccharomyces cerevisiae. J Bacteriol.

[CR6] Bozaquel-Morais BL, Madeira JB, Maya-Monteiro CM, Masuda Ca, Montero-Lomeli M (2010). A new fluorescence-based method identifies protein phosphatases regulating lipid droplet metabolism. PLoS ONE.

[CR7] Bredies K, Wolinski H (2011). An active-contour based algorithm for the automated segmentation of dense yeast populations on transmission microscopy images. Comput Vis Sci.

[CR8] Carman GM, Henry SA (2007). Phosphatidic acid plays a central role in the transcriptional regulation of glycerophospholipid synthesis in Saccharomyces cerevisiae. J Biol Chem.

[CR9] Carpenter AE, Jones TR, Lamprecht MR, Clarke C, Kang IH, Friman O, Guertin DA, Chang JH, Lindquist RA, Moffat J, Golland P, Sabatini DM (2006). Cell profiler: image analysis software for identifying and quantifying cell phenotypes. Genome Biol.

[CR10] Choi HS, Su WM, Morgan JM, Han GS, Xu Z, Karanasios E, Siniossoglou S, Carman GM (2011). Phosphorylation of phosphatidate phosphatase regulates its membrane association and physiological functions in Saccharomyces cerevisiae: identification of SER602, THR723, and SER744 as the sites phosphorylated by CDC28 (CDK1)-encoded cyclin-dependent kinase. J Biol Chem.

[CR11] Chumnanpuen P, Brackmann C, Nandy SK, Chatzipapadopoulos S, Nielsen J, Enejder A (2012). Lipid biosynthesis monitored at the single-cell level in Saccharomyces cerevisiae. Biotechnol J.

[CR12] Connerth M, Czabany T, Wagner A, Zellnig G, Leitner E, Steyrer E, Daum G (2010). Oleate inhibits steryl ester synthesis and causes liposensitivity in the yeast. J Biol Chem.

[CR13] Connerth M, Grillitsch K, Köfeler H, Daum G (2010). Analysis of lipid particles from yeast. Methods Mol Biol.

[CR14] Czabany T, Wagner A, Zweytick D, Lohner K, Leitner E, Ingolic E, Daum G (2008). Structural and biochemical properties of lipid particles from the yeast Saccharomyces cerevisiae. J Biol Chem.

[CR15] Dahlqvist A, Stahl U, Lenman M, Banas A, Lee M, Sandager L, Ronne H, Stymne S (2000). Phospholipid:diacylglycerol acyltransferase: an enzyme that catalyzes the acyl-CoA-independent formation of triacylglycerol in yeast and plants. Proc Natl Acad Sci USA.

[CR16] Ding Y, Zhang S, Yang L, Na H, Zhang P, Zhang H, Wang Y, Chen Y, Yu J, Huo C, Xu S, Garaiova M, Cong Y, Liu P (2013). Isolating lipid droplets from multiple species. Nat Protoc.

[CR17] Dirusso CC, Connell EJ, Faergeman NJ, Knudsen J, Hansen JK, Black PN (2000). Murine FATP alleviates growth and biochemical deficiencies of yeast fat1Delta strains. Eur J Biochem.

[CR18] Ejsing CS, Sampaio JL, Surendranath V, Duchoslav E, Ekroos K, Klemm RW, Simons K, Shevchenko A (2009). Global analysis of the yeast lipidome by quantitative shotgun mass spectrometry. Proc Natl Acad Sci USA.

[CR19] Evans CL, Xie XS (2008). Coherent anti-stokes Raman scattering microscopy: chemical imaging for biology and medicine. Annu Rev Anal Chem.

[CR20] Faber BC, Cleutjens KB, Niessen RL, Aarts PL, Boon W, Greenberg AS, Kitslaar PJ, Tordoir JH, Daemen MJ (2001). Identification of genes potentially involved in rupture of human atherosclerotic plaques. Circ Res.

[CR21] Faergeman NJ, Black PN, Zhao XD, Knudsen J, DiRusso CC (2001). The acyl-CoA synthetases encoded within FAA1 and FAA4 in Saccharomyces cerevisiae function as components of the fatty acid transport system linking import, activation, and intracellular utilization. J Biol Chem.

[CR22] Farese RV, Walther TC (2011). Lipid droplets finally get a little R-E-S-P-E-C-T. Cell.

[CR23] Fei W, Yang H (2012). Genome-wide screens for gene products regulating lipid droplet dynamics. Methods Cell Biol.

[CR24] Fei W, Shui G, Gaeta B, Du X, Kuerschner L, Li P, Brown AJ, Wenk MR, Parton RG, Yang H (2008). Fld1p, a functional homologue of human seipin, regulates the size of lipid droplets in yeast. J Cell Biol.

[CR25] Fei W, Shui G, Zhang Y, Krahmer N, Ferguson C, Kapterian TS, Lin RC, Dawes IW, Brown AJ, Li P, Huang X, Parton RG, Wenk MR, Walther TC, Yang H (2011). A role for phosphatidic acid in the formation of “supersized” lipid droplets. PLoS Genet.

[CR26] Fei W, Zhong L, Ta MT, Shui G, Wenk MR, Yang H (2011). The size and phospholipid composition of lipid droplets can influence their proteome. Biochem Biophys Res Commun.

[CR27] Fujimoto T, Ohsaki Y, Cheng J, Suzuki M, Shinohara Y (2008). Lipid droplets: a classic organelle with new outfits. Histochem Cell Biol.

[CR28] Garbarino J, Sturley SL (2009). Saturated with fat: new perspectives on lipotoxicity. Curr Opin Clin Nutr Metab Care.

[CR29] Garbarino J, Padamsee M, Wilcox L, Oelkers PM, D’Ambrosio D, Ruggles KV, Ramsey N, Jabado O, Turkish A, Sturley SL (2009). Sterol and diacylglycerol acyltransferase deficiency triggers fatty acid-mediated cell death. J Biol Chem.

[CR30] Gaspar ML, Jesch SA, Viswanatha R, Antosh AL, Brown WJ, Kohlwein SD, Henry SA (2008). A block in endoplasmic reticulum-to-golgi trafficking inhibits phospholipid synthesis and induces neutral lipid accumulation. J Biol Chem.

[CR31] Greenspan P, Fowler SD (1985). Spectrofluorometric studies of the lipid probe, Nile red. J Lipid Res.

[CR32] Greenspan P, Mayer EP, Fowler SD (1985). Nile red: a selective fluorescent stain for intracellular lipid droplets. J Cell Biol.

[CR33] Grillitsch K, Connerth M, Köfeler H, Arrey TN, Rietschel B, Wagner B, Karas M, Daum G (2011). Lipid particles/droplets of the yeast *Saccharomyces cerevisiae* revisited: lipidome meets proteome. Biochim Biophys Acta.

[CR35] Henry SA, Kohlwein SD, Carman GM (2012). Metabolism and regulation of glycerolipids in the yeast Saccharomyces cerevisiae. Genetics.

[CR34] Huh WK, Falvo JV, Gerke LC, Carroll AS, Howson RW, Weissman JS, O’Shea EK (2003). Global analysis of protein localization in budding yeast. Nature.

[CR36] Ivnitski-Steele I, Holmes AR, Lamping E, Monk BC, Cannon RD, Sklar LA (2009). Identification of Nile red as a fluorescent substrate of the Candida albicans ATP-binding cassette transporters Cdr1p and Cdr2p and the major facilitator superfamily transporter Mdr1p. Anal Biochem.

[CR37] Jacquier N, Schneiter R (2010). Ypk1, the yeast orthologue of the human serum- and glucocorticoid-induced kinase, is required for efficient uptake of fatty acids. J Cell Sci.

[CR38] Jacquier N, Choudhary V, Mari M, Toulmay A, Reggiori F, Schneiter R (2011). Lipid droplets are functionally connected to the endoplasmic reticulum in Saccharomyces cerevisiae. J Cell Sci.

[CR39] Jandrositz A, Petschnigg J, Zimmermann R, Natter K, Scholze H, Hermetter A, Kohlwein SD, Leber R (2005). The lipid droplet enzyme Tgl1p hydrolyzes both steryl esters and triglycerides in the yeast, *Saccharomyces cerevisiae*. Biochim Biophys Acta.

[CR40] Koffel R, Tiwari R, Falquet L, Schneiter R (2005). The Saccharomyces cerevisiae YLL012/YEH1, YLR020/YEH2, and TGL1 genes encode a novel family of membrane-anchored lipases that are required for steryl ester hydrolysis. Mol Cell Biol.

[CR41] Kohlwein SD (2000). The beauty of the yeast: live cell microscopy at the limits of optical resolution. Microsc Res Tech.

[CR42] Kohlwein SD (2010). Triacylglycerol homeostasis: insights from yeast. J Biol Chem.

[CR43] Kohlwein SD, Petschnigg J (2007). Lipid-induced cell dysfunction and cell death: lessons from yeast. Curr Hypertens Rep.

[CR44] Kohlwein SD, Veenhuis M, van der Klei IJ (2013). Lipid droplets and peroxisomes: key players in cellular lipid homeostasis or a matter of fat–store ‘em up or burn ‘em down. Genetics.

[CR45] Kurat CF, Natter K, Petschnigg J, Wolinski H, Scheuringer K, Scholz H, Zimmermann R, Leber R, Zechner R, Kohlwein SD (2006). Obese yeast: triglyceride lipolysis is functionally conserved from mammals to yeast. J Biol Chem.

[CR46] Le TT, Duren HM, Slipchenko MN, Hu CD, Cheng JX (2010). Label-free quantitative analysis of lipid metabolism in living Caenorhabditis elegant. J Lipid Res.

[CR47] Leber R, Zinser E, Zellnig G, Paltauf F, Daum G (1994). Characterization of lipid particles of the yeast, Saccharomyces cerevisiae. Yeast.

[CR48] Li H, Black PN, Chokshi A, Sandoval-Alvarez A, Vatsyayan R, Sealls W, DiRusso CC (2008). High-throughput screening for fatty acid uptake inhibitors in humanized yeast identifies atypical antipsychotic drugs that cause dyslipidemias. J Lipid Res.

[CR49] Li Z, Vizeacoumar FJ, Bahr S, Li J, Warringer J, Vizeacoumar FS, Min R, Vandersluis B, Bellay J, Devit M, Fleming JA, Stephens A, Haase J, Lin ZY, Baryshnikova A, Lu H, Yan Z, Jin K, Barker S, Datti A, Giaever G, Nislow C, Bulawa C, Myers CL, Costanzo M, Gingras AC, Zhang Z, Blomberg A, Bloom K, Andrews B, Boone C (2011). Systematic exploration of essential yeast gene function with temperature-sensitive mutants. Nat Biotechnol.

[CR50] Liang M-H, Jiang J-G (2013). Advancing oleaginous microorganisms to produce lipid via metabolic engineering technology. Prog Lipid Res.

[CR51] Lippincott-Schwartz J, Patterson GH (2003). Development and use of fluorescent protein markers in living cells. Science.

[CR52] Liu Q, Siloto RMP, Snyder CL, Weselake RJ (2011). Functional and topological analysis of yeast acyl-CoA:diacylglycerol acyltransferase 2, an endoplasmic reticulum enzyme essential for triacylglycerol biosynthesis. J Biol Chem.

[CR53] Malanovic N, Streith I, Wolinski H, Rechberger G, Kohlwein SD, Tehlivets O (2008). *S*-adenosyl-l-homocysteine hydrolase, key enzyme of methylation metabolism, regulates phosphatidylcholine synthesis and triacylglycerol homeostasis in yeast: implications for homocysteine as a risk factor of atherosclerosis. J Biol Chem.

[CR54] Matz MV, Fradkov AF, Labas YA, Savitsky AP, Zaraisky AG, Markelov ML, Lukyanov SA (1999). Fluorescent proteins from nonbioluminescent *Anthozoa* species. Nat Biotechnol.

[CR55] Miyanari Y, Atsuzawa K, Usuda N, Watashi K, Hishiki T, Zayas M, Bartenschlager R, Wakita T, Hijikata M, Shimotohno K (2007). The lipid droplet is an important organelle for hepatitis C virus production. Nat Cell Biol.

[CR56] Muller M, Zumbusch A (2007). Coherent anti-stokes Raman scattering microscopy. ChemPhysChem.

[CR57] Müllner H, Deutsch G, Leitner E, Ingolic E, Daum G (2005). YEH2/YLR020c encodes a novel steryl ester hydrolase of the yeast *Saccharomyces cerevisiae*. J Biol Chem.

[CR58] Murphy DJ (2001). The biogenesis and functions of lipid bodies in animals, plants and microorganisms. Prog Lipid Res.

[CR59] Murphy S, Martin S, Parton RG (2009). Lipid droplet-organelle interactions; sharing the fats. Biochim Biophys Acta.

[CR60] Natter K, Leitner P, Faschinger A, Wolinski H, McCraith S, Fields S, Kohlwein SD (2005). The spatial organization of lipid synthesis in the yeast *Saccharomyces cerevisiae* derived from large scale green fluorescent protein tagging and high resolution microscopy. Mol Cell Proteomics.

[CR61] Pagac M, de la Mora HV, Duperrex C, Roubaty C, Vionnet C, Conzelmann A (2011). Topology of 1-acyl-sn-glycerol-3-phosphate acyltransferases SLC1 and ALE1 and related membrane-bound *O*-acyltransferases (MBOATs) of *Saccharomyces cerevisiae*. J Biol Chem.

[CR62] Petschnigg J, Wolinski H, Kolb D, Zelling G, Kurat CF, Natter K, Kohlwein SD (2009). Good fat, essential cellular requirements for triacylglycerol synthesis to maintain membrane homeostasis in yeast. J Biol Chem.

[CR63] Rani SH, Saha S, Rajasekharan R (2013). A soluble diacylglycerol acyltransferase is involved in triacylglycerol biosynthesis in the oleaginous yeast *Rhodotorula glutinis*. Microbiology.

[CR64] Rockenfeller P, Ring J, Muschett V, Beranek A, Buettner S, Carmona-Gutierrez D, Eisenberg T, Khoury C, Rechberger G, Kohlwein SD, Kroemer G, Madeo F (2010). Fatty acids trigger mitochondrion-dependent necrosis. Cell Cycle.

[CR65] Sandager L, Gustavsson MH, Ståhl U, Dahlqvist A, Wiberg E, Banas A, Lenman M, Ronne H, Stymne S (2002). Storage lipid synthesis is non-essential in yeast. J Biol Chem.

[CR66] Schmidt C, Athenstaedt K, Koch B, Ploier B, Daum G (2013). Regulation of the yeast triacylglycerol lipase tgl3p by formation of nonpolar lipids. J Biol Chem.

[CR67] Schneiter R, Daum G (2006). Analysis of yeast lipids. Methods Mol Biol.

[CR68] Schneiter R, Daum G (2006). Extraction of yeast lipids. Methods Mol Biol.

[CR69] Schneiter R, Brügger B, Sandhoff R, Zellnig G, Leber A, Lampl M, Athenstaedt K, Hrastnik C, Eder S, Daum G, Paltauf F, Wieland FT, Kohlwein SD (1999). Analysis of the lipid molecular species composition of yeast subcellular membranes reveals acyl chain-based sorting/remodeling of distinct molecular species en route to the plasma membrane. Cell.

[CR70] Shaner NC, Campbell RE, Steinbach PA, Giepmans BN, Palmer AE, Tsien RY (2004). Improved monomeric red, orange and yellow fluorescent proteins derived from *Discosoma* sp. red fluorescent protein. Nat Biotechnol.

[CR71] Sitepu IR, Ignatia L, Franz AK, Wong DM, Faulina SA, Tsui M, Kanti A, Boundy-Mills K (2012). An improved high-throughput Nile red fluorescence assay for estimating intracellular lipids in a variety of yeast species. J Microbiol Methods.

[CR72] Snapp EL, Hegde RS, Francolini M, Lombardo F, Colombo S, Pedrazzini E, Borgese N, Lippincott-Schwartz J (2003). Formation of stacked ER cisternae by low affinity protein interactions. J Cell Biol.

[CR73] Spandl J, White DJ, Peychl J, Thiele C (2009). Live cell multicolor imaging of lipid droplets with a new dye, LD540. Traffic.

[CR74] Studer D, Humbel BM, Chiquet M (2008). Electron microscopy of high pressure frozen samples: bridging the gap between cellular ultrastructure and atomic resolution. Histochem Cell Biol.

[CR75] Szymanski KM, Binns D, Bartz R, Grishin NV, Li W-P, Agarwal AK, Garg A, Anderson RGW, Goodman JM (2007). The lipodystrophy protein seipin is found at endoplasmic reticulum lipid droplet junctions and is important for droplet morphology. Proc Natl Acad Sci USA.

[CR76] Taylor FR, Parks LW (1981). An assessment of the specificity of sterol uptake and esterification in Saccharomyces cerevisiae. J Biol Chem.

[CR77] Tong AH, Boone C (2006). Synthetic genetic array analysis in Saccharomyces cerevisiae. Methods Mol Biol.

[CR78] Tong AH, Evangelista M, Parsons AB, Xu H, Bader GD, Page N, Robinson M, Raghibizadeh S, Hogue CW, Bussey H, Andrews B, Tyers M, Boone C (2001). Systematic genetic analysis with ordered arrays of yeast deletion mutants. Science.

[CR79] Valachovič M, Hronská L, Hapala I (2001). Anaerobiosis induces complex changes in sterol esterification pattern in the yeast *Saccharomyces cerevisiae*. FEMS Microbiol Lett.

[CR80] Vizeacoumar FJ, Chong Y, Boone C, Andrews BJ (2009). A picture is worth a thousand words: genomics to phenomics in the yeast *Saccharomyces cerevisiae*. FEBS Lett.

[CR81] Vizeacoumar FJ, van Dyk N, Vizeacoumar FS, Cheung V, Li J, Sydorskyy Y, Case N, Li Z, Datti A, Nislow C, Raught B, Zhang Z, Frey B, Bloom K, Boone C, Andrews BJ (2010). Integrating high-throughput genetic interaction mapping and high-content screening to explore yeast spindle morphogenesis. J Cell Biol.

[CR82] Vorvis C, Markus SM, Lee WL (2008). Photoactivatable GFP tagging cassettes for protein-tracking studies in the budding yeast *Saccharomyces cerevisiae*. Yeast.

[CR83] Walther TC, Farese RV (2012). Lipid droplets and cellular lipid metabolism. Annu Rev Biochem.

[CR84] Wolinski H, Kohlwein SD (2008). Microscopic analysis of lipid droplet metabolism and dynamics in yeast. Methods Mol Biol.

[CR85] Wolinski H, Natter K, Kohlwein SD (2009). The fidgety yeast: focus on high-resolution live yeast cell microscopy. Methods Mol Biol.

[CR86] Wolinski H, Petrovič U, Mattiazzi M, Petschnigg J, Heise B, Natter K, Kohlwein SD (2009). Imaging-based live cell yeast screen identifies novel factors involved in peroxisome assembly. J Proteome Res.

[CR87] Wolinski H, Kolb D, Hermann S, Koning RI, Kohlwein SD (2011). A role for seipin in lipid droplet dynamics and inheritance in yeast. J Cell Sci.

[CR88] Wolinski H, Bredies K, Kohlwein SD (2012). Quantitative imaging of lipid metabolism in yeast: from 4D analysis to high content screens of mutant libraries. Methods Cell Biol.

[CR89] Wolinski H, Bredies K, Kohlwein SD (2012). Quantitative imaging of lipid metabolism in yeast: from 4D analysis to high content screens of mutant libraries. Methods Cell Biol.

[CR90] Yang HJ, Hsu CL, Yang JY, Yang WY (2012). Monodansylpentane as a blue-fluorescent lipid-droplet marker for multi-color live-cell imaging. PLoS ONE.

[CR91] Zechner R, Zimmermann R, Eichmann TO, Kohlwein SD, Haemmerle G, Lass A, Madeo F (2012). FAT SIGNALS–lipases and lipolysis in lipid metabolism and signaling. Cell Metab.

[CR92] Zinser E, Daum G (1995). Isolation and biochemical characterization of organelles from the yeast, *Saccharomyces cerevisiae*. Yeast.

[CR93] Zinser E, Paltauf F, Daum G (1993). Sterol composition of yeast organelle membranes and subcellular distribution of enzymes involved in sterol metabolism. J Bacteriol.

[CR94] Zweytick D, Athenstaedt K, Daum G (2000). Intracellular lipid particles of eukaryotic cells. Biochim Biophys Acta.

[CR95] Zweytick D, Leitner E, Kohlwein SD, Yu C, Rothblatt J, Daum G (2000). Contribution of Are1p and Are2p to steryl ester synthesis in the yeast *Saccharomyces cerevisiae*. Eur J Biochem.

